# Intergenerational Ambivalence, Loneliness, and Well-Being Among Older Adults in the United States

**DOI:** 10.1093/geroni/igab046.2107

**Published:** 2021-12-17

**Authors:** Xiaoyan Zhang, Merril Silverstein

**Affiliations:** 1 Syracuse University, Syracuse, New York, United States; 2 Syracuse University, Syracuse, New York, New York, United States

## Abstract

Intergenerational relationships are important sources of informal social support for older people to maintain their emotional well-being. Previous research has extensively investigated the relationship between intergenerational support and older adult’s psychological well-being. However, the existing research has not adequately examined intergenerational ambivalence – mixed or contradictory feelings toward a family member in another generation or explored the mechanism that links intergenerational ambivalence and psychological well-being. Further, most studies are cross-sectional, which prevents us from establishing causality. This study utilized data from 2006, 2008, 2010, 2012, 2014, and 2016 waves of Health and Retirement Study (HRS), a national representative sample of U.S. adults aged 50 and more (N= 8,017). Structural equation models were used to examine the longitudinal relationship between intergenerational ambivalence, loneliness, depression, and life satisfaction. The final model indicated very good fit (χ2 = 113.31, p < .0001, CFI = 0.97, RMSEA = .05). The results revealed that ambivalence in older parent-adult child relationships predicted higher subsequent loneliness (β = 0.21, p < .0001), which in turn predicted depressive symptoms (β = 0.25, p <.0001) and life satisfaction (β = -0.30, p < .0001). The results demonstrated that loneliness mediated the relationship between intergenerational ambivalence and depression, and life satisfaction. Multiple group analysis was performed to test whether the study relationships varied by gender. Gender differences were found. Findings have implications for prevention and intervention initiatives targeting improving relationships between parents and children, thereby protecting against mental problems.

